# Coexpression of Spectrally Distinct Rhodopsins in *Aedes aegypti* R7 Photoreceptors

**DOI:** 10.1371/journal.pone.0023121

**Published:** 2011-08-08

**Authors:** Xiaobang Hu, Michelle A. Whaley, Michelle M. Stein, Bronwen E. Mitchell, Joseph E. O'Tousa

**Affiliations:** Department of Biological Sciences, University of Notre Dame, Notre Dame, Indiana, United States of America; New Mexico State University, United States of America

## Abstract

The retina of the mosquito *Aedes aegypti* can be divided into four regions based on the non-overlapping expression of a UV sensitive Aaop8 rhodopsin and a long wavelength sensitive Aaop2 type rhodopsin in the R7 photoreceptors. We show here that another rhodopsin, Aaop9, is expressed in all R7 photoreceptors and a subset of R8 photoreceptors. In the dorsal region, Aaop9 is expressed in both the cell body and rhabdomere of R7 and R8 cells. In other retinal regions Aaop9 is expressed only in R7 cells, being localized to the R7 rhabdomere in the central and ventral regions and in both the cell body and rhabdomere within the ventral stripe. Within the dorsal-central transition area ommatidia do not show a strict pairing of R7–R8 cell types. Thus, Aaop9 is coexpressed in the two classes of R7 photoreceptors previously distinguished by the non-overlapping expression of Aaop8 and Aaop2 rhodopsins. Electroretinogram analysis of transgenic *Drosophila* shows that Aaop9 is a short wavelength rhodopsin with an optimal response to 400–450 nm light. The coexpressed Aaop2 rhodopsin has dual wavelength sensitivity of 500–550 nm and near 350 nm in the UV region. As predicted by the spectral properties of each rhodopsin, *Drosophila* photoreceptors expressing both Aaop9 and Aaop2 rhodopsins exhibit a uniform sensitivity across the broad 350–550 nm light range. We propose that rhodopsin coexpression is an adaptation within the R7 cells to improve visual function in the low-light environments in which *Ae. aegypti* is active.

## Introduction

Visual input is critical to the behavior of *Ae. aegypti* and other mosquito species [1], [2] that are the vectors for many pervasive and devastating tropical diseases. In adult mosquitoes, visual information is acquired by the compound eye, an organized array of ∼300–400 identical units called ommatidia. Each ommatidium has eight photoreceptor cells (R1–R8), each possessing a light sensitive organelle called the rhabdomere. The outer R1–R6 photoreceptors project rhabdomeres inward to form a fused rhabdom structure surrounding the central R8 cell. The R8 photoreceptor projects a rhabdomere outward that contacts the R1 rhabdomere. The R7 cell body is located between two outer photoreceptors while its rhabdomere is positioned at the top of the fused rhabdom [3], [4].

Rhodopsins are G-protein coupled receptors embedded in rhabdomere membranes that initiate visual transduction. Animal genomes typically contain multiple rhodopsin genes with different spectral properties such that the expression of different rhodopsins in distinct classes of photoreceptor cells provides the basis for color vision. There are 10 predicted rhodopsin genes in the *Ae. aegypti* genome [5]. They are classified into five different groups on the basis of sequence similarity with *Drosophila* and other invertebrate rhodopsins [5]. These are a long wavelength group (λmax >500 nm) of five members, the short wavelength (λmax 400–500 nm) Aaop9, the UV sensitive (λmax <400 nm) Aaop8, and the two poorly characterized groups represented by Aaop10 and the pteropsin, Aaop12. This large family of rhodopsins is also present in *Anopheles* and *Culex* genomes, suggesting a conserved use of visual information in the behavioral strategies of these mosquitoes.

The identification of the photoreceptor cell type expressing each of these rhodopsins is needed to understand the organization of the mosquito retina and the mechanisms involved in the processing of visual information. In the *Drosophila* retina, there are two major classes of ommatidia based on the pairing of the R7 and R8 cells. These ommatidia either express rhodopsin Rh3 in the R7 cell and Rh5 in the R8 cell, or express Rh4 in the R7 cell and Rh6 in the R8 cell. This rhodopsin pairing is mediated by a signal from the R8 cell to the R7 cell [6]. Previously, we showed that the UV sensitive Aaop8 rhodopsin and a long wavelength sensitive Aaop2 rhodopsin are expressed in non-overlapping subsets of the *Ae. aegypti* R7 photoreceptor cells [3]. Phylogenetic analysis shows that the *Ae. aegypti* Aaop9 rhodopsin is the closest relative of the *Drosophila* Rh5 rhodopsin expressed in a subset of R8 cells [5], [7]. We show here that Aaop9 is expressed in a retinal pattern that is distinct from the *Drosophila* model.

Visual systems typically benefit from the use of multiple rhodopsins, each with distinct spectral properties. Typically, a photoreceptor will express a single rhodopsin to serve as the basis of color discrimination [8] although exceptions are now known in both vertebrates and invertebrates [9], [10]. In this report we show that the Aaop9 rhodopsin is coexpressed with other rhodopsins in *Ae. aegypti* R7 photoreceptor cells. Notably, coexpression with the long wavelength rhodopsin Aaop2 provides the basis for these R7 photoreceptor cells to respond across a broad spectrum of visible and UV light. We discuss the potential of this adaptation for acquiring visual information from the low light environments in which mosquitoes are active.

## Materials and Methods

### Detection of *Ae. aegyptii* Aaop9 and other rhodopsin proteins

The peptide corresponding to the N-terminal 5 through 19 amino acids of the *Ae. aegypti* Aaop9 rhodopsin (CNETDAAIFPMARTGD) was chemically synthesized with a cysteine added at the 5′ end to allow for conjugation to KLH. The peptide was then conjugated to KLH and used to immunize two rabbits and the sera were affinity purified by a commercial supplier (Biomatik, Ontario, Canada). In addition, the Aaop9 peptide was conjugated to KLH by using the Imject Maleimide Activated mcKLH Kit (Pierce, Rockford, IL) and mice were immunized using Titer Max Gold Adjuvant (Sigma, St. Louis, MO) to produce mouse anti-Aaop9 polyclonal antibody. The Aaop9 mouse antibody showed the same specificity as the Aaop9 rabbit antibody. The Institutional Animal Care and Use Committee at the University of Notre Dame approved the mouse immunization protocol (Protocol Number 11-010). Immunizations were carried out by the Freimann animal care facility's technicians at Notre Dame using their standard operating procedure to assure adherence to appropriate guidelines for ethical animal use. The production of Aaop8 mouse polyclonal antibody and Aaop2 rabbit polyclonal antibody was described previously [3].

For protein blots, *Ae. aegypti Kh^w^* strain heads and bodies were homogenized in 1× lysis buffer (30 mM Tris-HCl, pH 6.8, 10% SDS, 0.0002% bromophenol blue, 5% β-mercaptoethanol, 10% glycerol). Protein from two *Ae. aegypti* heads or one body were loaded and fractionated on a NuPAGE Novex 4–12% Bis-Tris gel (Invitrogen) and transferred to PVDF membrane. Membranes were probed with 1∶3000 dilution of Aaop9 antiserum at 4°C overnight, detected by using horseradish peroxidase-linked goat anti-rabbit IgG (1∶2000, GE Healthcare) and developed with the ECL Western Blotting Detection System (GE Healthcare) according to the manufacturer's protocol.

For preparation of retinas for whole mounted observation, adult mosquito heads (white-eyed *Kh^w^* strain) were bissected, leaving one eye undamaged, and fixed overnight with 2% paraformaldehyde in PBS at 4°C. Retinas were dissected, washed three times in PBT (1×PBS/0.1% Tween-20), and incubated with the anti-Aaop9 polyclonal antiserum (1∶100) and/or anti-Aaop8 polyclonal antiserum (1∶100) diluted in BNT (1× PBS/0.1% BSA/0.1% Triton/250 mM NaCl) 12–18 h at 4°C. After three times 10 min wash with PBT, retinal tissues were incubated with fluorescently labeled secondary antibodies (1∶500 diluted in BNT) for 2 h at RT. After 3 times 10 min washes with PBT, retinal tissues were then mounted in Vectashield (Vector Laboratories). The secondary antibodies used were the Alexa Fluor 488 goat anti-rabbit secondary antibody (1∶500 diluted in BNT), Alexa Fluor-594 goat anti-mouse secondary antibody (1∶500 diluted in BNT), and DyLight™-649 donkey anti-mouse (Jackson ImmunoResearch). Alexa Fluor 594 phalloidin (1∶40) (Molecular Probes, Carlsbad, CA) was used to label actin.

To prepare retinal sections, mosquito heads and *Drosophila* heads were cut and fixed in 4% paraformaldehyde/5% sucrose overnight at 4°C, rinsed 3 times 10 min in 5% sucrose/1× PBS, placed in 5% sucrose/1× PBS overnight at 4°C, then placed in 30% sucrose/1× PBS overnight at 4°C, and finally in 30% sucrose/1× PBS∶Tissue Freezing Medium (1∶1; Triangle Biomedical Sciences) for 4 h at RT. Tissues were then embedded and frozen in 100% Tissue Freezing Medium and sectioned at 10∼12 µm. Slides were dried at 50°C for 2 h. Sections were rehydrated with 1× PBS for 20 min, placed in blocking buffer (1× PBS/2.5% normal goat serum/0.3% Triton X-100/1% DMSO) for 1 h, and incubated overnight at 4°C with rabbit or mouse anti-Agop9 (1∶100 dilution) and mouse anti-Aaop8 or rabbit anti-Aaop2 (1∶100 dilution) polyclonal antisera. After three 10 min washes in PBT, samples were incubated in goat anti-rabbit or -mouse Alexa Fluor 488 (1∶500) and goat anti-mouse or -rabbit Alexa Fluor 594 (1∶500) diluted in blocking buffer for 1 h at room temperature. Sections were washed three times 10 min in PBT, 5 min in PBS, and then mounted using Vectashield. Confocal microscopy was used to image both whole mounted and sectioned retinas.

### Transgenic *Drosophila* expressing *Ae. aegypti* Aaop9 and Aaop2 rhodopsins in R1–6 photoreceptors

The Aaop2 and Aaop9 cDNA were characterized in the *Ae. aegypti* genome project (clones NADBM43 and NABXH41 respectively) and provided by the laboratory of Dr. David Severson at University of Notre Dame. Both ORFs were directionally cloned into a modified pCaSpeR4 expression vector in which the polylinker region was replaced with the *Drosophila* Rh1 gene (*ninaE*) promoter, an EcoR1-Not1 cloning site, and a 0.7 kb 3′ untranslated region of the *ninaE* gene [11] to place the Aaop9 and Aaop2 ORFs under the control of the *Drosophila* Rh1 promoter. Transgenic *Drosophila* strains carrying each of the transgenes in a Rh1 null (*ninaE^I17^*) genetic background were generated by standard techniques [11], [12]

ERG analysis was carried out using standard procedures [13]. All flies were made white-eyed by introducing a 2^nd^ chromosome containing *cn bw* into the genetic background. The flies also contained genetic elements that eliminated the ERG response from the R7 and R8 photoreceptors [11]. To gauge spectral responses, narrow bandpass filters (Newport, Irvine, CA) at 600 nm, 550 nm, 500 nm, 450 nm, 400 nm and 350 nm were sequentially positioned in the light path of a 1000 W tungsten light source (Oriel, Irvine, CA). Stimuli were monitored by a high resolution spectrometer (Ocean Optics Model HR2000CG-UV-NIR) and equalized to within 8% with neutral density filters. An optical power meter (Newport Model 840) determined that radiance at all wavelengths was approximately 600 µW/cm^2^.

## Results

### Aaop9 rhodopsin is expressed in both R7 and R8 photoreceptors in the dorsal region and only R7 photoreceptors in the ventral stripe

**Figure 1:**
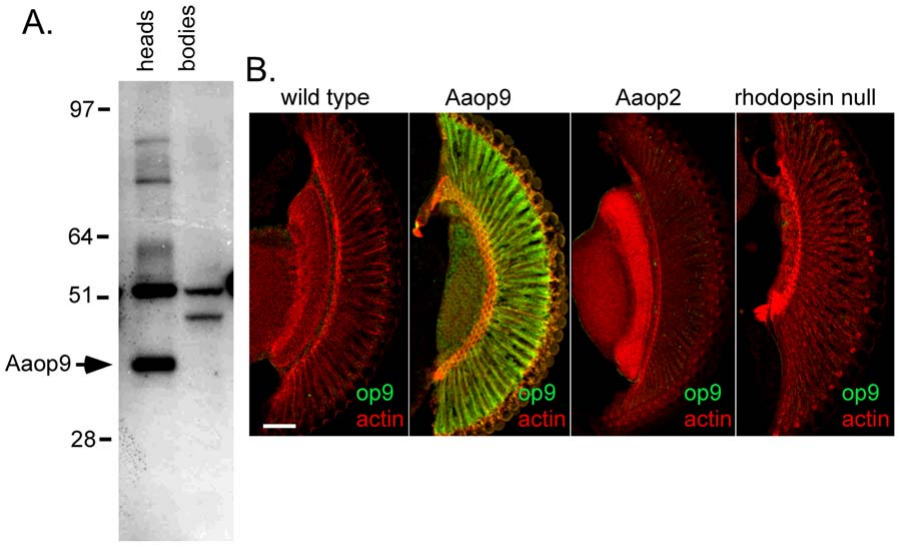
Specificity of *Ae. aegypti* Aaop9 antisera. A. Protein blot analysis shows the *Ae. aegypti* Aaop9 antiserum recognizes a 39 kD protein in *Ae. aegypti* heads but not bodies. Some higher molecular weight bands only detected in the head sample may identify Aaop9 rhodopsin multimers; a non-related crossreacting protein at 45–50 kD is found in both head and body samples. B. Immunofluorescent detection of *Ae. aegypti* Aaop9 expression in transgenic *Drosophila* shows Aaop9 expression in R1–6 photoreceptor cells (middle left panel) have strong retinal labeling, while minimal labeling is seen in retinal sections (other panels) of *Drosophila* expressing Rh1 rhodopsin (wild type), *Ae. aegypti* Aaop2 rhodopsin, or no rhodopsin (rhodopsin null). All flies were in a white eyed genetic background. The scale bar shown in panel at right is ∼50 µm.

To determine the expression of Aaop9 rhodopsin in the *Ae. aegypti* eye, we generated polyclonal antisera against a peptide corresponding to the N-terminal 5–19 amino acids of Aaop9 protein. This sequence is unique to Aaop9 and is not expected to cross react with other *Ae. aegypti* rhodopsins. Protein blot analysis showed that this Aaop9 antiserum recognized a protein in *Ae. aegypti* heads, but not in bodies, of approximately 39 kDa (arrowhead), near the expected 43 kD size of the Aaop9 rhodopsin (Figure 1A). To confirm the specificity of the antiserum, we examined transgenic *Drosophila* expressing Aaop9 or other *Ae. aegypti* rhodopsins. Fig. 1B shows that the retinas of wild-type non-transgenic flies and transgenic flies expressing Aaop2 rhodopsin fail to stain with the Aaop9 antiserum while strong staining was evident in the transgenic flies expressing Aaop9.

**Figure 2:**
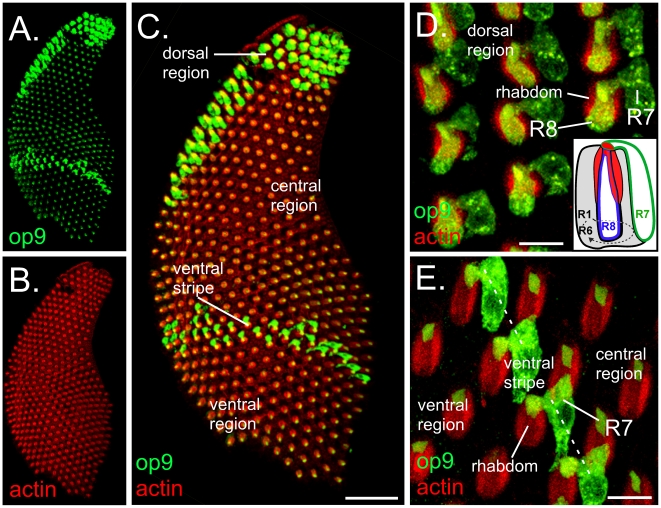
Aaop9 rhodopsin is expressed in all R7 and a subset of R8 photoreceptor cells in *Ae. aegypti*. A–C. Whole mount retina of *Ae. aegypti* stained with Aaop9 antibody (A, green), phalloidin (B, red), and the resulting merged image (C). Phalloidin detects actin and heavily stains the fused rhabdom of all ommatidia. Aaop9 shows different expression profiles in the regions of the *Ae. aegypti* retina labeled as the dorsal region, central region, ventral stripe, and ventral region in C. The scale bar shown in C is ∼50 µm. D. A magnified view of the dorsal region of the *Ae. aegypti* retina. In one ommatidium the rhabdom and the R7 and R8 cell bodies are identified. Aaop9 rhodopsin (green) is expressed in the R8 cell positioned centrally within the rhabdom and in the R7 cell projecting a rhabdomere at the distal surface of the rhabdom. The inset drawing shows a single ommatidial unit oriented in a similar fashion as those in the micrograph. The fused rhabdom is shown in red, the R7 cell (green) projects a distal rhabdomere and the R8 cell (blue) occupies the central area inside the rhabdom. The R1–6 cell bodies (grey) surround the rhabdom. In this dorsal region, Aaop9 rhodopsin is localized to the cell bodies and rhabdomeres of the R7 and R8 photoreceptors. The scale bar shown is ∼10 µm. E. A magnified view of the ventral stripe region of the *Ae. aegypti* retina. In one ommatidum within the ventral stripe the R7 cell body is identified. In the central and ventral regions on either side of the ventral stripe, Aaop9 rhodopsin (green) is expressed only in the R7 rhabdomere. Within the ventral stripe, Aaop9 rhodopsin is expressed in R7 cell body (labeled) and the R7 rhabdomere. The rhabdom (red) is labeled; the orientation of ommatidia is similar to the inset drawing shown in Fig. 2D. Scale bar is ∼10 µm.

To determine the expression pattern of the Aaop9 rhodopsin in *Ae. aegypti*, we carried out immunostaining analysis of whole-mounted retina. Fig. 2A–C shows a nearly complete retinal preparation stained for Aaop9 (green) and actin (red). Actin is highly enriched in the microvillar rhabdomeres of retinal photoreceptors and provides a convenient marker for the ommatidial organization of the retina. There are major differences in Aaop9 rhodopsin expression within different regions of the retina. Four regions are recognized as labeled in Fig. 2C: the dorsal region, the central region, the ventral stripe, and the ventral region. These are the same four regions previously identified by the non-overlapping expression pattern of Aaop8 and Aaop2 type rhodopsins in the R7 photoreceptor cells [3].

**Figure 3:**
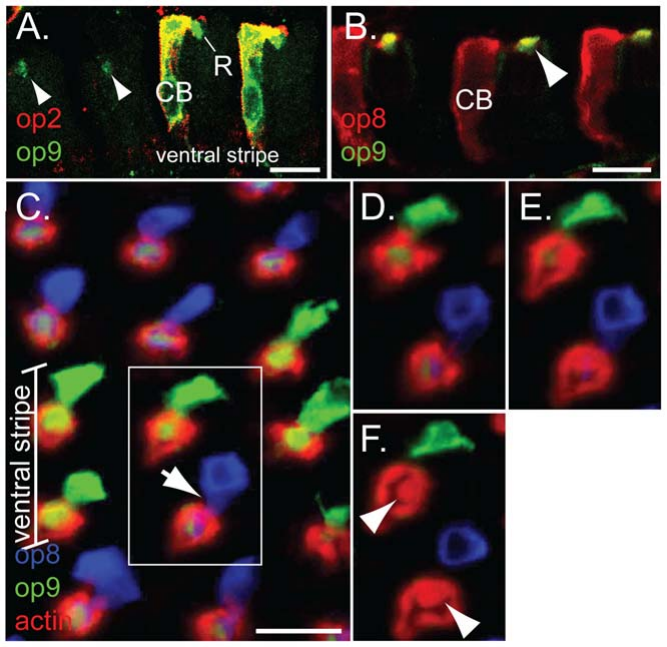
Aaop9 rhodopsin expression pattern in the ommatidia of central, ventral stripe, and ventral regions. A. An *Ae. aegypti* retinal section including the ventral stripe showing longitudinal views of Aaop2-expressing R7 cells (red). These cells also express Aaop9 (green) within the cell body (CB) and rhabdomere (R). Aaop9 is also weakly detected at discrete sites in the central region (arrowheads). These sites are documented in Fig. 3B to be R7 cell rhabdomeres. The double labeling was carried out using a mouse Aaop9 antibody as described in Materials and Methods. All scale bars are ∼10 µm. B. A retinal section of *Ae. aegypti* showing longitudinal views of Aaop8-expressing R7 cells (red) in the central region. Aaop9 (green) is localized to the rhabdomere of these R7 cells. C. Aaop8, Aaop9, and actin localization in x, y confocal microscope views of whole mounted retina in the ventral stripe region. The boxed region shows ommatidia viewed at a depth of the R7 rhabdomere (furthest distal), deduced from the presence of a stalk (arrow) connecting the R7 cell body to the rhabdom. D, E, F. Additional x, y optical sections of the two ommatidia boxed in C. These sections are 1 µm above, 1 µm below, and 3 µm below, respectively, of the section displayed in C. Aaop9 is not detected in the proximal section (F) that shows the R8 rhabdomere (arrowheads) as well as the outer (R1–6) rhabdomeres.

The dorsal region in *Ae. aegypti* retina is defined by Aaop2 type rhodopsin expression within the R7 photoreceptor [3]. A high magnification view of ommatidial units within the dorsal region showed that Aaop9 is expressed within the rhabdomere and cell bodies of both the R7 and R8 photoreceptors (Fig. 2D). Aaop9 also localizes to the R7 cell body and rhabdomere in the ventral stripe (Fig. 2E). To confirm that the ventral stripe Aaop9 expression corresponds to those ommatidia expressing Aaop2, retinal sections were colabeled with both antibodies. A retinal section showing a longitudinal view of colabeled photoreceptors near the ventral stripe is shown in Fig. 3A. This experiment showed that in the ventral stripe, all cells showing Aaop2 expression also show Aaop9 expression within both the cell body (CB) and rhabdomere (R). The low level of Aaop9 expression outside the ventral stripe (arrowhead) is analyzed in the next section.

### Aaop9 is expressed in the R7 cells of the central and ventral regions

Within the central and ventral regions, Aaop9 is expressed at a lower level and is confined to the rhabdomeric region of a central photoreceptor cell type (Fig. 2C, 2E). To identify this central cell type, we co-labeled the retina with Aaop8 (expressed in the R7 cells in the central and ventral regions) and Aaop9 antibodies. Fig. 3B shows a sectioned retina with Aaop9 and Aaop8 costaining in ommatidial units within the central region. This image shows Aaop8 localization (red) in the rhabdomere (apical projection, marked with an arrow in middle ommatidium) as well as in the cell bodies (CB) of the R7 cells. Aaop9 is colocalized with Aaop8 within the R7 cell rhabdomere, but, unlike Aaop8, Aaop9 cannot be detected in the R7 cell body.

The R8 cell rhabdomere lies directly below the R7 rhabdomere within the center of the rhabdom [4]. To determine if Aaop9 is also localized to the rhabdomeres of R8 cells, whole mount retina were triple stained for Aaop8, Aaop9 and actin. Fig. 3C shows a confocal section of a distal retinal region containing the ventral stripe. This view shows that within the ventral stripe, Aaop9 is expressed in both the cell body and rhabdomere of R7 cell. Outside of the stripe, corresponding to the central and ventral regions, Aaop9 expression is limited to the center of the fused rhabdom which could represent the R7 or R8 rhabdomere. This is likely the R7 rhabdomere in all these ommatidia because the stalk connecting the R7 cell body to the rhabdom is visible (labeled in the ommatidium by an arrow). This stalk is present only in the most distal part of rhabdom where the R7 photoreceptor projects a rhabomere over the fused rhabdom. Hence these results are consistent with those in Fig. 3B showing that Aaop9 is expressed in the rhabdomeres of Aaop8 expressing R7 cells in the central and ventral regions. Fig. 3D–F provide additional evidence showing different focal planes of the boxed region in Fig. 3C, representing sections 1 µm distal, 1 µm proximal and 3 µm proximal to the section shown in Fig. 3C. Aaop9 and Aaop8 expression is found in the R7 cells of the distal sections (Fig. 3D,E). In the proximal section shown in Fig. 3F, the R8 rhabdomeres are visible in the center region of the fused rhabdom (arrows). There is no Aaop8 and Aaop9 signal in the rhabdom, confirming that Aaop9 expression is limited to the R7 rhabdomere in the central and ventral regions.

### Mixed Aaop9 expression pattern is present at the dorsal-central transition area

**Figure 4:**
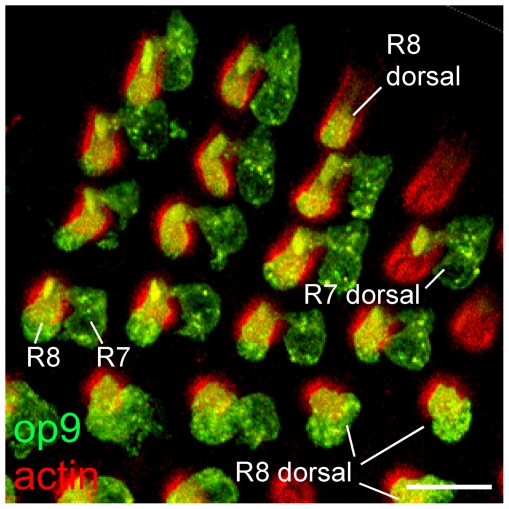
Mixed Aaop9 expression pattern at the dorsal-central transition area of the *Ae. aegypti* retina. Whole mount image of ommatidia located in the transition area between the dorsal and central region. Ommatidia on the left side are located in the dorsal region and express Aaop9 (green) in both R7 and R8 cell body and rhabdomere. One ommatidium showing Aaop9 rhodopsin expression (green) in the R7 cell but not the R8 cell is marked as “R7 dorsal”, and several ommatidia showing Aaop9 rhodopsin expression in R8 cell but not the R7 cell are marked as “R8 dorsal”. Actin is stained by phalloidin (red) to identify the rhabdom of all ommatidia. The orientation of ommatidia is similar to the inset drawing shown in Fig. 2D. The scale bar is ∼20 µm.

As shown in Fig. 2A–D, in the dorsal retina, Aaop9 is localized to both the rhabdomere and the cell body of R7 and R8 cells. However, in the transition area from the dorsal to central regions, there are ommatidia with unpaired R7 and R8 cells. As shown in Fig. 4, one ommatidium (marked as “R7 dorsal”) has Aaop9 cell body expression in the R7 cell but not in the R8 cell. The reciprocal is also observed, in which Aaop9 is expressed in the R8 cell, but not in the R7 cell body (marked as “R8 dorsal”). Ommatidia with unpaired R7 and R8 cell expression of Aaop9 suggest that one central cell type is not signaling to direct rhodopsin expression in the other central cell type cell within this region of the retina.

### Analysis of Aaop9 and Aaop2 spectral properties in transgenic flies

**Figure 5:**
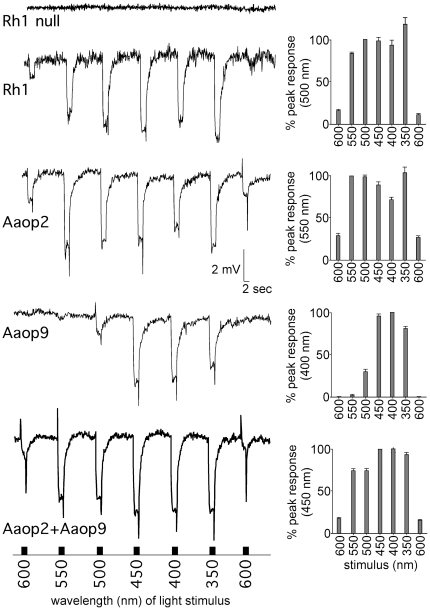
Spectral analysis of *Ae. aegypti* Aaop9 and Aaop2 rhodopsins in transgenic *Drosophila*. ERG recordings displaying light response elicited from *Drosophila* R1–6 cells in *ninaE^I17^* (Rh1 null), Rh1 (wild type), and transgenic *Drosophila* expressing Aaop2, Aaop9, or both Aaop2 and Aaop9. Two-second light pulses of intensity-equalized lights at 600 nm, 550 nm, 500 nm, 450 nm, 400 nm, 350 nm, and 600 nm were administered at 16 seconds intervals. ERG traces showing a typical response for each genotype is on the left and graphs showing average responses to each wavelength is on the right. Average responses (mean and SEM, sample sizes Rh1 (n = 5), Aaop2 (n = 4), Aaop9 (n = 5), and Aaop2+Aaop9 (n = 4)) are calculated relative to the peak response to visible light. The UV peak for Rh1 (118%) and Aaop2 (104%) exceeded the visible light peak response. The complete genotypes of the flies were Rh1 null: *w norpA^P24^*/Y; *cn bw*; <pRh1-*norpA*> *ninaE^I17^*/<pRh1-*norpA*> *ninaE^I17^*, Rh1: *w norpA^P24^*/Y; *cn bw*; <pRh1-*norpA*> *ninaE^I17^*/+, Aaop2: *w norpA^P24^*/Y; *cn bw*; <pRh1-*norpA*> *ninaE^I17^*/<pRh1-Aaop2> *ninaE^I17^*, Aaop9: *w norpA^P24^*/Y; <pRh1-Aaop9> *cn bw/cn bw*; <pRh1-*norpA*> *ninaE^I17^*/*ninaE^I17^*, and Aaop2+Aaop9: *w norpA^P24^*/Y; <pRh1-Aaop9> *cn bw/cn bw*; <pRh1-*norpA*> *ninaE^I17^*/<pRh1-Aaop2> *ninaE^I17^*.

To study the spectral properties of the *Ae. aegypti* Aaop9 and Aaop2 rhodopsins, we placed Aaop9 and Aaop2 cDNAs under the control of the *Drosophila ninaE* (Rh1) gene promoter. These gene constructs were introduced into *ninaE^I17^* (Rh1 null) flies to allow expression of the mosquito rhodopsins in the *Drosophila* R1–6 photoreceptor cells. Two additional genetic manipulations were made so that the electroretinogram (ERG) response would be generated exclusively from the R1–6 cells. First we created a null *norpA^P24^* mutant background and then placed the *norpA* ^+^ gene under control of the Rh1 promoter into this genetic background. This provides phospholipase C expression, and hence a photoresponse, only from the R1–6 cells. In Fig. 5, the top trace shows this genetic background, in the absence of a transgenically expressed rhodopsin (Rh1 null), effectively eliminated the ERG response for all light stimuli. The second trace documents recovery of the ERG response when the *Drosophila* Rh1 gene is introduced into this genetic background.

Fig. 5 also shows the ERG responses for the Aaop2, Aaop9, and Aaop2+Aaop9 expressing flies. These responses were recorded in white-eyed flies at 600, 550, 500, 450, 400, and 350 nm. Each light stimulus was attenuated with neutral density filters to bring all stimuli to 0.030±0.002 lux in order to determine the relative sensitivity of the rhodopsin to each wavelength. The control Rh1 trace (Fig. 5, second trace) shows this experimental approach accurately identifies the two peak sensitivities for the *Drosophila* Rh1 rhodopsin, one between 450–500 nm corresponding to direct Rh1 activation, and a second UV peak near 350 nm due to energy transfer from a UV-sensitizing pigment [14], [15]. The third trace shows that *Ae. aegypti* Aaop2 rhodopsin also exhibits dual wavelength sensitivity. The long wavelength peak resides between 500–550 nm, and a second peak is found in the UV region near 350 nm.

The ERG analysis of the flies expressing *Ae. aegypti* Aaop9 rhodopsin (Fig. 5, fourth trace) reveals that Aaop9 rhodopsin possesses a single spectral sensitivity peak within the 400–450 nm range. The fifth trace shows flies expressing both Aaop9 and Aaop2 rhodopsins possess broad spectral sensitivity, with strong responses at all wavelengths from 350 to 550 nm.

## Discussion

In *Drosophila*, the R7 and R8 photoreceptor cells are distinguished by their positioning within the ommatidial unit and their expression of distinct classes of rhodopsins. Due to these properties, these two cell classes have important roles in color vision and the detection of polarized light [16]. Further, the R7 and R8 cells are matched in *Drosophila*, such that an ommatidium expressing Rh5 in the R8 cell will express Rh3 in the R7 cell and an ommatidium expressing Rh6 in the R8 cell will express Rh4 in the R7 cell [6], [17]. This creates a mosaic of Rh3/Rh5 and Rh4/Rh6 units intermixed throughout most of the *Drosophila* retina.

The placement of the R7 and R8 cells and their rhabdomeres is strikingly different in *Ae. aegypti* than in *Drosophila*. In *Ae. aegypti*, the patterned expression of two rhodopsins in the R7 cell creates well organized retina, with defined dorsal, central, ventral stripe, and ventral regions [3]. The R7 cell elaborates a rhabdomere only at the distal surface of the fused rhabdom. The R8 photoreceptor cell body is located inside the ommatidium and the R8 cell is the only cell to project a rhabdomere outwardly into the fused rhadom [3].

In the work here we have characterized the expression pattern of the *Ae. aegypti* Aaop9 rhodopsin. This rhodopsin possesses the highest level of sequence identity with Rh5, a rhodopsin expressed in one class of *Drosophila* R8 photoreceptors. To determine which photoreceptors express Aaop9 in *Ae. aegypti*, 15 amino acids within the N terminal domain unique to the Aaop9 protein was used to create a polyclonal antibody. Protein blots showed the antiserum detected a 39 kD protein in *Ae. aegypti* heads, and this protein is not present in *Ae. aegypti* bodies. Immunostaining of transgenic *Drosophila* expressing different *Ae. aegypti* rhodopsins also confirmed the specificity of the Aaop9 antibody.

Application of this antibody in both whole mount and sectioned retina preparations established that Aaop9 has a very unique expression pattern in *Ae. aegypti* retina. In the dorsal region, Aaop9 is expressed in both the rhabdomere and cell body of R7 and R8 cells. An abundance of rhodopsin within the cell body was previously observed for the Aaop2 and Aaop8 rhodopsins expressed in the different classes of *Ae. aegypti* R7 cells [3]. Rhodopsin localization within cytoplasmic compartments has been best documented for *Limulus*, in which movement of rhodopsin into the cytoplasm is light-triggered and serves as the mechanism for desensitization [18]. Further studies are needed to determine if similar processes occur in mosquitoes.

Aaop9 is expressed in R7 cells throughout the retina but its expression in the R8 cells is restricted to the dorsal region. This expression pattern was unexpected because Aaop9's closest relative in *Drosophila* is Rh5, a R8 cell-specific rhodopsin expressed in ∼40% of ommatidia throughout the retina [19]. These results, along with our earlier report [3], document the extensive differences in retinal organization and rhodopsin expression patterns between *Drosophila* and mosquitoes.

### Specifying the identity of R7 and R8 cells within the dorsal region

*Drosophila* achieves the pairing of R7 and R8 rhodopsin expression by cell signaling during development [6]. Here we show that the dorsal region of the *Ae. aegypti* retina pairs R7 and R8 rhodopsin expression that is distinct from that of the other regions. However, based on the analysis of Aaop9 expression in the dorsal-central transition area, it is unlikely that R7–R8 cell communication is responsible for the arrangement. We observed that within the transition area some ommatidia possess a R7 cell with the dorsal type of rhodopsin expression while the R8 cell is a central type lacking Aaop9 rhodopsin expression. The reciprocal mixed ommatidia are also observed in which the R7 cell possesses the central, while the R8 cell the dorsal, type of Aaop9 rhodopsin expression. These results are not expected based on a model of R7 cell-R8 cell signaling as described in *Drosophila* [20] but rather is consistent with a model in which a developmental cue is highly expressed in the dorsal region and decreases in a ventral directed gradient. The Iroquois complex genes are reported to form this type of gradient in the *Drosophila* eye [21]. If *Ae. aegypti* R7 and R8 cells are individually responding to a gradient developmental signal, it would lead to the formation of the dorsal-central transition region where the R7 and R8 cells of an ommatidium do not always make the same dorsal versus central decision.

### Coexpression of Aaop9 and Aaop2 rhodopsins in the R7 photoreceptors of the dorsal and ventral stripe regions

The dorsal region and ventral stripe of the *Ae. aegypti* eye were originally identified by the expression of Aaop2 within the R7 cells [3]. In the current work we show that these R7 cells also express Aaop9. It is rare that a single receptor cell expresses more than one rhodopsin protein, but this does occur in some vertebrates and invertebrates [10]. To investigate the physiological significance of Aaop2 and Aaop9 coexpression, we characterized the spectral responses of these two rhodopsins in transgenic *Drosophila*. Both rhodopsins were capable of producing light responses in *Drosophila*, which is anticipated as transgenic *Drosophila* has been successfully used to express the rhodopsins of even more distantly related invertebrates such as honeybees [22] and horseshoe crabs [23].

ERG analysis of transgenic *Drosophila* showed that *Ae. aegypti* Aaop2 has a peak sensitivity between 500–550 nm. This confirms that Aaop2 is a long wavelength rhodopsin as inferred from phylogenetic analysis [5], [24]. Aaop2 also shows a second peak of sensitivity in the UV region near 350 nm. UV peak sensitivity has been documented in long wavelength rhodopsins of other Dipterans, and is due to the transfer of light energy from a UV sensitizing pigment to the long wavelength rhodopsin [25]. ERG analysis showed that Aaop9 is a short wavelength rhodopsin with maximal spectral sensitivity at approximately 400 nm. This result is also in agreement with expectations from phylogenetic comparisons [5].

Coexpression of Aaop9 and Aaop2 in *Drosophila* R1–6 photoreceptor cells provides a broadband sensitivity extending from 350 nm to 550 nm. The mechanism of broadening spectral sensitivity by dual rhodopsin expression was described previously in the *Papilio* butterfly [26]. Our results suggest that coexpression of these two rhodopsins in *Ae. aegypti* increases the spectral range of the R7 photoreceptor at the expense of color discrimination. One consideration is the capacity of mosquito rhodopsin to couple to the *Drosophila* phototransduction machinery. A bias favoring one rhodopsin will reduce the other rhodopsin's input towards the spectral response, and result in some color filtering. While color filtering has been described in insect eyes [27], none of the identified color filtering molecules are rhodopsin proteins. A second consideration is that perhaps each rhodopsin couples to a separate G protein and subsequent transduction machinery, as observed in a vertebrate lizard [28]. Neither of these possibilities seems likely in *Ae. aegypti*. First, the ERG analysis showed that Aaop2 and Aaop9 couple effectively to one *Drosophila* G protein, and the mosquito genome contains only one corresponding G protein ortholog [5]. Second, the use of light filters will reduce sensitivity, which is a disadvantage for an organism active in dim light conditions. It is difficult to determine if the Aaop2∶Aaop9 expression ratio and their respective activities in transgenic *Drosophila* approximates the situation in *Ae. aegypti*. In any case, the presence of the second rhodopsin, given the documented differences in spectral sensitivity, will diminish color discrimination.

### Aaop9 in R7 photoreceptors of the central and ventral regions

The R7 photoreceptors of the central and ventral regions were previously characterized as expressing the UV sensitive Aaop8 rhodopsin [3]. We showed here that these R7 cells also express Aaop9, but the expression profile is different in two respects from the Aaop9 expression profile in the dorsal and ventral stripe regions. First, the immunofluorescence data suggest Aaop9 is present at a much lower concentration in these cells. Second, Aaop9 is localized only to the rhabdomeric region of the Aaop8-expressing R7 cells and not within the cell body. The reason for this distinction is not known and will require further investigation. Aaop8 is a UV rhodopsin, responding maximally at 350 nm and lacking any response within the visible wavelength range (data not shown). As Aaop9 has a peak sensitivity in the 400–450 range and appears to be expressed at a much lower level than the coexpressed Aaop8, Aaop9 may only have a small effect on the spectral sensitivity of these central and dorsal R7 cells.

### The significance of rhodopsin coexpression in R7 photoreceptors

*Ae. aegypti* and many other mosquito species are active in low light environments. In these environments, the effectiveness of photon capture is key to the acquisition of useful visual information. In principle, photon capture can be improved by (1) increasing the concentration of rhodopsin within the photosensitive membranes, (2) increasing the surface area of the light sensitive membranes, (3) increasing the efficiency of photon capture, and (4) minimizing filtering loss of photons prior to photon capture. The type of rhodopsin expressed is not expected to alter rhodopsin concentration or the photosensitive surface area. The third possibility, increasing the efficiency of photon capture, can be achieved by coexpression of rhodopsins with different spectral properties. The positioning of the R7 rhabdomere at the apical surface of the ommatidial unit is also a key consideration. This location minimizes the possibility of light loss due to absorption or reflection as light passes through other biological material. Also, the wide aperture of a mosquito lens provides superior light gathering but limits resolution to a “blur circle” at a specific depth below the lens [29]. Placing the R7 rhabdomere at this depth in dark-adapted animals allows the R7 cells to have an optimal chance of acquiring useful visual information when vision is limited by low light. Thus, considering the limitations imposed by the primary design and the size constraints of the mosquito compound eye, rhodopsin coexpression in R7 cells is one viable adaptation for enhancing mosquito vision in low light environments.
